# Abu Bakr Muhammad Ibn Zakariya Al-Razi (Rhazes) (865-925): The Founder of the First Psychiatric Ward

**DOI:** 10.7759/cureus.64601

**Published:** 2024-07-15

**Authors:** Jaafar O Ahmed, Karwan K Kakamad, Zana B Najmadden, Sarhang I Saeed

**Affiliations:** 1 Psychology, Soran University, Soran, IRQ; 2 Research, Kscien Organization, Sulaymaniyah, IRQ; 3 Research, University of Halabja, Halabja, IRQ; 4 Clinical Psychology, Koya University, Sulaymaniyah, IRQ

**Keywords:** psychotherapy, medieval ages, al-razi, psychiatric hospital, historical vignette

## Abstract

Abu Bakr Muhammad Ibn Zakariya Al-Razi, also known as Rhazes, was a 10th-century Persian polymath who made significant contributions to medicine, philosophy, chemistry, and psychiatry. He is credited with founding the first psychiatric ward in Baghdad, highlighting the medical treatment of mental illnesses. His empirical and innovative approaches to clinical observation and experimentation laid the basis for modern evidence-based medicine. Al-Razi's comprehensive works, such as "The Comprehensive Book," profoundly influenced both Islamic and European medical practices, securing his legacy as a pivotal figure in medical history.

Therefore, the primary objective of this narrative review is to revisit the remarkable contributions of Al-Razi in the field of psychiatry, specifically highlighting his role as the founder of the first psychiatric ward.

## Introduction and background

Abu Bakr Muhammad Ibn Zakariya Ibn Yahya Al-Razi, also known as Rhazes in the Western world [1-4], was a Persian polymath [5]. Al-Razi was one of the most famous and respected physicians of the 10th century, and he was celebrated for his groundbreaking contributions to medicine and psychiatry [6,7]. He has been celebrated as "the greatest physician of Islam and the Medieval Ages" [4,6], as noted by George Sarton in his "Introduction to the History of Science" [3]. He is renowned for his profound contributions to medicine, chemistry, philosophy, and psychiatry. Al-Razi's intellectual pursuits and innovative methodologies have secured his legacy as one of the most influential figures in the history of medicine [8,9]. His innovative works in various fields established the foundation for modern evidence-based medicine [6].

Al-Razi's influence and authority in medicine remained unrivaled until the 17th century [3]. However, his most significant and unique contribution was in the realm of mental health. He is credited with establishing the first psychiatric ward, a testament to his progressive understanding of mental illness as a medical condition requiring systematic and compassionate treatment [1-3]. Al-Razi's significance in the medical world is highlighted by his empirical approach to medicine, a method that emphasizes the importance of clinical observation, experimentation, and integrating different medical traditions [6]. This rigorous approach allowed him to challenge and improve upon the established medical knowledge of his time, making significant advancements in various medical disciplines [6,8,9].

He was a distinguished scientist whose influence extended beyond the Muslim world to Europe as well [10]. He authored over 200 books, with half focusing on medicine and others covering philosophy, theology, mathematics, astronomy, and chemistry [11]. His pioneering works earned him numerous titles, such as "the Arab Galen" [10], the father of Islamic medicine [12], the "greatest physician of the medieval period" [11], a "prominent medical scientist of the middle ages" [10], the greatest Arabic-Islamic physician [5], the father of pediatrics [8], a physician for all seasons [13], and the encyclopedist [14]. In May 1970, the Bulletin of the World Health Organization paid special tribute to Al-Razi, highlighting his original and accurate writings on smallpox and measles, and noting his essay on infectious diseases as the first scientific treatise on the subject [14,15]. Thus, Al-Razi was an exceptional clinician, teacher, researcher, and original thinker [13].

The main aim of this narrative review is to explore and highlight the pioneering contributions of Al-Razi to the field of mental health and psychiatry by examining his works in mental health and the establishment of the first psychiatric ward.

## Review

Al-Razi's life and career

He was born in 865 AD (251 Hegira) in Ray and died in 925 AD [5]. His lifespan has been variously described as 846-930 [8] and 854-932 [16]. However, we have adopted the period from the most frequently cited references as 865-925 [1,4-7,9,11-13]. Ray is approximately 20 miles south of modern Tehran, Iran [6]. In his early years, he pursued various interests, including music and chemistry. Before ultimately focusing on medicine at around 30 years old [5,11], he was a skillful lute player [10]. He developed an interest in medicine at the age of 30 when he visited the renowned Azudi Hospital in Baghdad [14]. His quest for knowledge led him to study in various Islamic educational institutions, where he emerged as a distinguished scholar renowned for his intellectual curiosity and extensive knowledge. He was well-versed in the ancient Greek language [15]. Al-Razi traveled extensively, journeying to renowned medical hubs of his era such as Jerusalem, Cairo, and Cordova [1,16]. During his youth, he moved to Baghdad, pursuing medical studies and later practicing at a local hospital [7].

Al-Razi's career was marked by several prestigious appointments. He served as the chief physician and dean of medical school at the Baghdad Hospital, one of the most advanced medical centers of its time, and his reputation as an exceptional doctor proliferated [1,17]. Throughout his career, Al-Razi authored more than 200 books on a wide range of subjects, showcasing his vast knowledge and intellectual versatility [6,7]. In his later life, Al-Razi returned to Ray, where he gathered many students around him [10].

**Figure 1:**
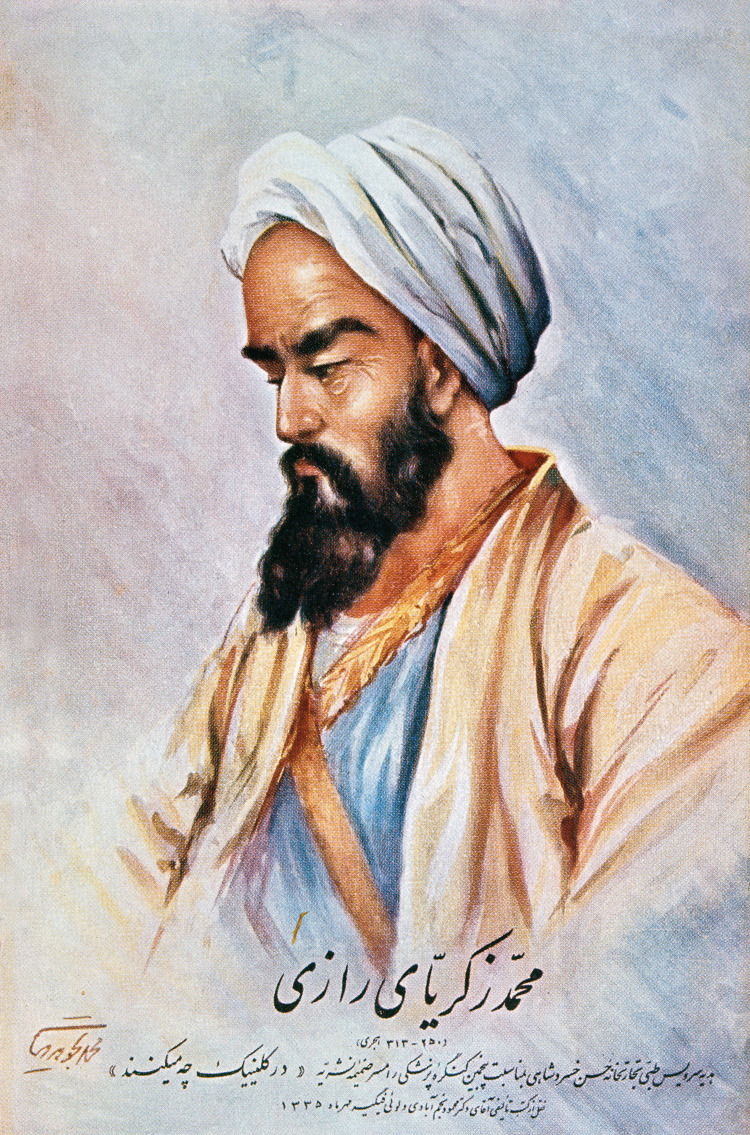
Portrait of Al-Razi (Rhazes) (AD 865-925) Reproduced with permission from Wellcome Collection [19]

Despite his fame, Al-Razi never amassed wealth, as he often treated poor patients without demanding payment. As a result, he lived in relative poverty [3,10]. His contributions to medicine are still honored in his homeland, where his birthday (August 27) is celebrated annually as National Iranian Pharmacists' Day [9]. In his later years, he developed cataracts in both eyes and became blind [8]. When confronted with the need for eye surgery, Al-Razi rejected a surgeon who had not mastered the anatomy of the eye [9]. He passed away in Ray on October 27, 925, at 60 (Figure 1) [5,6,18].

Influences on Al-Razi

Al-Razi was influenced by a wide array of scholars and previous works. The medical knowledge of ancient Greece and Rome, particularly the writings of Hippocrates and Galen, significantly shaped his medical philosophy. The intellectual environment of the Islamic Golden Age, characterized by the translation and preservation of ancient texts, provided a rich foundation for Al-Razi's studies and allowed him to access and synthesize a wide range of medical knowledge [6]. A seminal feature of his genius was his acknowledgment of the wisdom of the Greeks and Romans [12]. However, he did not merely accept their teachings; he critically analyzed and often corrected their theories based on his observations and experiments [8,9]. In his book "Shukuk 'ala Alinusor" (Doubts About Galen), Al-Razi expressed his gratitude to Galen for his commendable contributions, stating, "It grieves me to oppose and criticize the man Galen, from whose sea of knowledge I have drawn much. Indeed, he is the Master, and I am the disciple. However, this reverence and appreciation will and should not prevent me from doubting, as I did, what is erroneous in his theories" [9]. Under the guidance of the well-known philosopher Abu Zaid Ahmad Ibn Sahl Al-Balkhi, Al-Razi delved into philosophy and formulated his philosophical framework [3].

His teacher in medicine was Ali Ibn Sahl Rabban Al-Tabari (838-870), a physician and philosopher born in Merv, Tabaristan of modern-day Iran [2,9,15]. Al-Tabari, originally Persian, was a trailblazer in child development, as outlined in his book "Firdaus al-Hikmah." Firdaus is a comprehensive medical text structured into seven sections and 30 treatises [2]. Tabari was regarded as his mentor [15]. Al-Razi immersed himself in the study of medicine and quite likely delved into philosophy under the tutelage of Ibn Rabban at the Muqtadari Hospital, where he grew practical knowledge and later became its head [2,5,15]. Additionally, he received instruction from a disciple of Hunayn Ibn Ishaq, who possessed deep knowledge of ancient Greek, Persian, and Indian medical traditions, among other subjects [3].

Al-Razi's books

He wrote more than 200 books on different topics, including medicine, philosophy, chemistry, physics, astronomy, theology, and music [5,7], of which about 40 have persisted [1,7,10]. A significant portion of his writings were lost [10], and many of his books were plagiarized [18]. Several of his works were translated into Latin, French, Italian, Hebrew, and Greek [13]. Approximately 40 of his medical books and treatises are still extant in the libraries and museums of Iran, Britain, France, India, and the Library of Congress of the United States [15]. In a paper about the bibliography of the works of Al-Razi, Deuraseh (2008) [18] enumerates 184 books, including 56 books on medicine, 33 on natural sciences, seven on logic, 10 on mathematics and astronomy, seven commentaries, abridgments, and summaries, 17 on philosophical and hypothetical sciences, six on metaphysics, four on the divine sciences, 22 on chemistry, two on heretical books, and 10 on the various arts [18]. Table 1 shows the most famous books with a short description of each.

**Table 1:** Al-Razi's famous writings This table outlines important books written by Al-Razi, adapted from various scholarly sources [2,5-10,12].

Arabic name	English name	Description
Kitab al-Hawi	The Comprehensive Book	Compilation of Al-Razi's readings, clinical observations, case studies, and treatments
Kitab al-Mansuri Fi al-Tibb	The Book of Al-Mansur in Medicine	Concise medical handbook written for Abu Salih Al-Mansur in 903
Kitab al-Judari wa al-Hasbah	The Book of Smallpox and Measles	Discusses differentiation and treatment of smallpox and measles
Mujarabbat	The Book of Experiences	A book on hospital experiences
Kitab al-Murshid	The Guide	Medical handbook providing guidance on diagnosis, treatment, and management of illnesses
Kitab al-Shukuk 'ala alinusor	Doubts About Galen	Critiques Galen's theories, presenting Al-Razi’s clinical observations
Man la Yahduruhu Al-Tabib	For One Without a Doctor	Practical handbook of home remedies for travelers and the poor
Risāla fi amrāz al-atfāl wa ‘I- ‘ianaya bihim	A Treatise on Pediatric Disease	Covers various illnesses in newborns, infants, and children
Kitab Būr’ al-Sā’ah	Cure in an Hour	Remedies for ailments believed to be curable within an hour
Al-Tibb Al-Ruhani	Spiritual Medicine	Methods for treating moral and psychological afflictions
Kitab Daf' Madarr al-Aghiyah	Book on Repelling Harms Caused by Diets	Encyclopedia of foods, detailing health benefits and avoiding negative effects
Kitab al-Hasa fi al-kula wa al-mathanah	Stones in the Kidney and Bladder	Detailed examination of stone formation in kidneys and bladder
Kitab fi sifat al bimaristan	Book on the Characteristics of the Hospital	Discusses necessary characteristics of a hospital
Akhlaq-Al-Tabib	Medical Ethics	Emphasizes ethical obligations of physicians to patients and vice versa

Through translation, his medical works became famous among medieval European practitioners and significantly influenced medical education in the West [6]. His most important work is the medical encyclopedia known as Al-Hawi fi al-Tibb, known in Europe as Liber Continens [8,9]. Al-Razi's medical writings were characterized by their clarity, thoroughness, and practical relevance. His works were widely translated and disseminated, influencing medical practice in the Islamic world and medieval Europe [3,5]. "Kitab al-Judari wa al-Hasbah" was translated more than a dozen times into Latin [5]. Some sections of his publications were integrated into the medical curriculum at Western universities [6,13]. During the 15th and 16th centuries, his writings became essential teaching texts in European medical schools [11,15]. In 1395, al-Hawi was one of the nine volumes constituting the entire library of the Paris Faculty of Medicine. Furthermore, Max Meyerhof published translations of 33 of the surviving case histories from the Continens [3]. Al-Razi's Latin-translated books are preserved in the libraries of Munich, Paris, and the Escorial [10].

Al-Razi's works in the field of medicine

**Figure 2:**
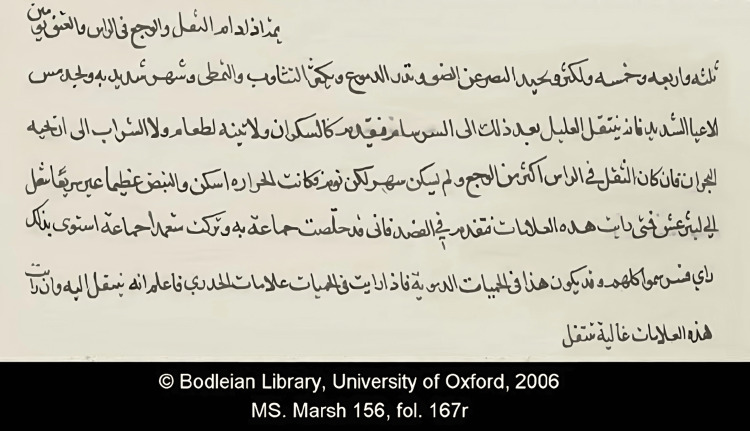
A page from The Comprehensive Book (Arabic edition) Translation of the text: "When the dullness (thiqal) and the pain in the head and neck continue for three and four and five days or more, and the vision shuns light, and watering of the eyes is abundant, yawning and stretching are great, insomnia is severe, and extreme exhaustion occurs, then the patient after that will progress to meningitis (sirsâm) … If the dullness in the head is greater than the pain, and there is no insomnia, but rather sleep, then the fever will abate, but the throbbing will be immense but not frequent and he will progress into a stupor (lîthûrghas). So when you see these symptoms, then proceed with bloodletting. For I once saved one group [of patients] by it, while I intentionally neglected [to bleed] another group. By doing that, I wished to reach a conclusion (ra'y). And so all of these [latter] contracted meningitis." Reproduced with permission from The James Lind Library [21]

Al-Razi's contributions to medicine were vast and varied, encompassing numerous fields and advancing the understanding of various diseases, including pediatrics, neurology, psychosomatic medicine, pharmacology, and medical ethics [4-7]. His magnum opus, "Kitab al-Hawi" (The Comprehensive Book), was one of the most comprehensive medical texts of its time. This encyclopedia detailed various diseases, their symptoms, and treatments, drawing from a wide range of sources and incorporating Al-Razi's clinical observations and experiments (Figure 2) [8,20].

Al-Razi made significant contributions to neurology [7], neuroanatomy [5,9], and neurosurgery [9]. He identified nerves as having motor or sensory functions, detailing seven cranial nerves and 31 spinal cord nerves. He adeptly combined his knowledge of cranial and spinal nerve anatomy with clinical observations to accurately localize lesions in the nervous system [3,5,9]. Additionally, he is recognized as the first physician to differentiate and identify concussion as a distinct neurological condition from others [5]. He is recognized as the first person to identify and describe the laryngeal branch of the recurrent laryngeal nerve [3].

Al-Razi was also known for his pioneering works in the field of infectious diseases, particularly smallpox and measles; he is considered the "original portrayer" of smallpox [6,9,14]. In his treatise "On Smallpox and Measles," he differentiated between the two diseases for the first time, providing detailed clinical descriptions and suggesting treatments based on the severity of the symptoms [9]. Also, Al-Razi was the first to recognize allergic rhinitis. In a monograph on the subject, he described a patient experiencing attacks of coryza, rhinitis, and sneezing whenever roses were in bloom [3,12]. These works were highly influential and remained a reference point for many years, highlighting Al-Razi's contributions to epidemiology and infectious disease management [9].

Furthermore, Al-Razi made significant contributions to pharmacology [8]. He compiled extensive lists of medical substances and their uses, documenting the properties and effects of various drugs [5]. His empirical approach to pharmacology led to the discovery of new treatments and the development of medicinal compounds, including the introduction of mercurial ointments [5,8]. He declared the therapeutic features of opium [22] and suggested the use of opium as an anesthetic [3]. He also developed various instruments used in apothecaries, such as mortars and pestles, flasks, spatulas, beakers, and glass vessels, which were used in pharmacies till the early 20th century [8].

His emphasis on clinical observation, experimentation, and empirical research set new standards in medical practice and significantly advanced the understanding and treatment of diseases [3,9]. For example, Al-Razi used the differential diagnosis approach to evaluate his patients, which remains a cornerstone of modern medical practice [9]. One of his well-known achievements was in determining a good place for hospitals [14]. An often-recounted anecdote describes how Al-Razi determined the location of a hospital in Baghdad. According to the legend, Al-Razi was tasked with selecting the ideal site for the hospital. To make his decision, he hung pieces of fresh meat at various potential construction sites around the city. After some time, he checked the meat and chose the site where the meat had putrefied the slowest, indicating it as the best location for the hospital [15,17]. He is regarded as a physician who practiced evidence-based medicine early in medical history [6]. Without the sophisticated diagnostic tests available to modern physicians, Al-Razi devised clever and ingenious methods for diagnosis. For instance, he diagnosed diabetes by instructing a suspected patient to urinate on the sand. If ants gathered on the spot after a while, it indicated that the patient had diabetes [13]. He is a pioneer in many areas of medicine.

Al-Razi made significant contributions to various areas of medicine. He was the first to observe the pupillary reaction to light and described cataract surgery, stating, "I have split the lower part of the pupil and have led the cataract outward" [15]. In his important monograph "Stones in the Kidney and Bladder," he discussed in detail the formation of stones in the kidneys and bladder [10,12]. Al-Razi used the term "sudden death" in Arabic 1000 years ago, highlighting the heart's role in syncope and sudden death [13]. In "Akhlaq-Al-Tabib" (Medical Ethics), he outlined rules for both physicians and patients to consider [23]. Additionally, he differentiated rheumatism from gout in his writings [11].

Al-Razi and mental health foundations

Al-Razi's approach to mental health was revolutionary for his time. He viewed mental illnesses as conditions that required medical intervention, challenging the prevalent notions that attributed such ailments to supernatural causes or moral failings [2,20]. His understanding of psychology and psychiatry was advanced. Considering the influence of emotions and psychological well-being on physical health, Al-Razi combined psychological techniques with physiological explanations [1,3]. Al-Razi explored concepts of reinforcement, reward, and punishment; for instance, he differentiated between internal and external forms of positive reinforcement in the learning of new behaviors and manners [24]. Similar to modern psychologists such as Bowlby, who developed attachment theory, Al-Razi recognized the significant impact of the parent-child relationship, early training, and child-rearing practices on the development of a healthy personality in adulthood [25].

He is a valued source of motivation in the history of psychiatry [7]. In his medical writings, Al-Razi was a principal of psychosomatic medicine [2]. He advocated for a humane and ethical approach to treatment, highlighting the need for a supportive environment for patients [1,4,16]. Al-Razi gave priority to the doctor-patient relationship [10]. He advised physicians on how to keep the respect and confidence of their patients [1]. His holistic approach to medicine reflected this perspective, which integrated physical, psychological, and environmental factors [7]. He also treated his clients with respect, care, and empathy [1]. Common aspects of their therapy include focusing on the client-therapist relationship, self-analysis, exchanging feedback, self-reflection, reasoning, self-awareness, and having the courage to be genuine [25].

His books provided explanations for various mental illnesses that afflicted society during the 10th century. He also outlined the symptoms, definitions, differential diagnoses, and treatments for different mental illnesses [7]. For example, conditions such as schizophrenia were distinguished from manic-depressive psychoses [19]. Al-Razi, in his renowned medical book Kitab al-Hawi, wrote a chapter addressing mental illness (Gunun), in which he observed that a confusional state (Ikhtilat) often followed febrile and physical illness. He distinguished between melancholia and Gunun (madness), noting that a person suffering from Gunun is agitated and persistently mentally confused, with a complete loss of reason, whereas in melancholia, the reason is merely misdirected. He also clarified that a Majnun (insane) is not epileptic, as an epileptic person is otherwise healthy except during seizures [20].

Al-Razi advocated for psychotherapy [1,2,7,16,26]. He emphasized that positive remarks from doctors could uplift patients, enhance their well-being, and facilitate a faster recovery. Al-Razi also believed that a sudden, intense emotional reaction could rapidly improve psychological, psychosomatic, and organic disorders [2]. He used psychotherapy in a simple but dynamic approach [3,7]. He also asserted that religious compulsions could be overcome by reason to achieve better mental health [2], so he accomplished a primary form of cognitive therapy for obsessive behavior [7]. Besides religious and cognitive therapy [25], Al-Razi also used music therapy [9,17,24].

He recognized mental health and self-esteem as crucial factors influencing a person's overall health and well-being. Embracing the idea of "a sound mind in a healthy body," he successfully aided many patients in achieving complete health [7,25]. He posited that chronic intrapsychic conflicts could lead to various physical disorders, as described in his work Al-Tibb Al-Ruhani [25]. For instance, he proposed that "even if the physician harbors doubts, they should always instill hope in the patient, as the mind's state affects the body's condition" [11]. To treat Prince Mansur, the governor of Ray, for a rheumatic or psychosomatic condition causing severe joint and back pain that hindered his movement, Al-Razi resorted to psychotherapy, as traditional medicine had failed. He requested the fastest horse and a mule and then took Prince Mansur to a Turkish hot bath outside the city, where he had the horse saddled with the prince's belongings for an escape. Without knowing Al-Razi's plan, the prince relaxed alone in the bath. Suddenly, Al-Razi brandished a large dagger, angrily confronting Prince Mansur about his soldiers' insolence in forcing Al-Razi to treat him. Feigning a murderous rage, Al-Razi threatened the unguarded prince. Overcome with fear and anger, Prince Mansur leaped up and fled on the horse. Al-Razi later wrote to the prince, congratulating him on his recovery and explaining the therapeutic intent behind his staged outburst [2,16]. Table 2 recaps Al-Razi's contributions to mental health.

**Table 2:** Al-Razi's contributions to mental health This table summarizes the works of Al-Razi in mental health, adapted from various scholarly sources [1-5,7-11,16,22,23].

Category	Details
Historical significance	First to treat mental illness as a medical condition rather than a supernatural or moral failing
Psychological innovations	Advanced understanding of the role of emotions, psychological well-being, and the parent-child relationship in mental health
Concepts explored	Reinforcement, reward, punishment, and the impact of early relationships on personality development
Medical philosophy	Advocated a humane and ethical approach to mental health treatment, emphasizing the doctor-patient relationship and a holistic perspective
Approach to treatment	Holistic, integrating physical, psychological, and environmental factors
Key publications	Kitab al-Hawi: discussed mental illness, symptoms, and treatments
Therapeutic methods	Psychotherapy, cognitive therapy, music therapy, and emotional reaction strategies
Philosophical views	Mental health as essential for overall well-being; promoted the idea of "a sound mind in a healthy body"
Notable case study	Treatment of Prince Mansur using innovative psychological methods
Significant achievements	Established the first psychiatric ward in Baghdad, marking a pioneering advancement in institutional mental healthcare

Al-Razi as the founder of the first psychiatric ward

One of Al-Razi's most significant achievements was the establishment of the first psychiatric ward in Baghdad [1,3,5,7,16,24]. The history of care for the mentally ill reflects human cultural diversity; the earliest known mental hospitals were established in the Islamic world, with notable institutions in Baghdad (918 CE) and Cairo [20,27]. At that time, hospitals, known as Samaritans (bimaristan means a place where patients reside) had separate wards for various ailments, including medicine, surgery, fever, wounds, mania, and eye diseases [17]. The hospitals cared for both violent insane individuals and chronic cases of mental illness [20]. These Islamic psychiatric wards gained renown for their compassionate and "moral" approach to treatment, with a notable focus on inclusion rather than isolation [24]. During this period, European institutions lacked similar facilities due to prevailing fears of demonic possession [16]. In contrast, throughout the Golden Age of Islam, esteemed hospitals were established in major cities across the Muslim world, including Damascus, Alexandria, and Cairo [24].

In 907, he was employed as a director of a large hospital in Baghdad [1,8]. While serving as the director of a hospital in Baghdad, Al-Razi introduced the concept of psychiatric wards dedicated to the care of patients with mental illness. He advocated that mental disorders should be recognized and treated as medical conditions [5,7]. At those wards, Al-Razi conducted thorough clinical observations of patients with psychiatric conditions and implemented treatment strategies involving diet, medication, occupational therapy, aromatherapy, baths, and music therapy [7]. These wards are also famous for hosting the idea of the psychiatric milieu: providing patients with clean clothes, bathing, persistent activities, and a healthy diet [24]. As part of discharge planning, patients were given money to support their immediate needs and facilitate their reintegration into society. This marks one of the earliest recorded instances of psychiatric aftercare in history [7,17]. Also distinctive was the interdisciplinary composition of the treatment teams, which, in addition to physicians, frequently included roles akin to modern nurses, social workers, chaplains, and pharmacists. This holistic approach aimed at addressing the entire patient rather than merely their medical condition [24].

The establishment of the psychiatric ward had a lasting impact on the development of mental healthcare. It set new standards for the treatment of mental health conditions and provided a model for subsequent institutions. Al-Razi's work in this area laid the groundwork for modern psychiatry and the integrated approach to mental healthcare that is used today.

## Conclusions

Al-Razi's efforts significantly influenced psychiatry and medicine. His creative treatments, particularly in mental health, and empirical methodology established the groundwork for contemporary evidence-based medicine. He contributed to the advancement of organized and humane treatment for mental disorders by founding the first psychiatric ward in Baghdad. Al-Razi had an impact on both Islamic and Western medicine.
